# Heterostructural transformation of mesoporous silica–titania hybrids

**DOI:** 10.1038/s41598-020-80584-8

**Published:** 2021-02-05

**Authors:** Navarut Paengjun, Kasimanat Vibulyaseak, Makoto Ogawa

**Affiliations:** School of Energy Science and Engineering, Vidyasirimedhi Institute of Science and Technology (VISTEC), Rayong, 21210 Thailand

**Keywords:** Photocatalysis, Porous materials, Composites, Nanoparticles

## Abstract

Mesoporous silica (SBA-15 with the BJH pore size of 8 nm) containing anatase nanoparticles in the pore with two different titania contents (28 and 65 mass%), which were prepared by the infiltration of the amorphous precursor derived from tetraisopropyl orthotitanate into the pore, were heat treated in air to investigate the structural changes (both mesostructure of the SBA-15 and the phase and size of the anatase in the pore). The mesostructure of the mesoporous silica and the particle size of anatase unchanged by the heat treatment up to 800 °C. The heat treatment at the temperature higher than 1000 °C resulted in the collapse of the mesostructure and the growth of anatase nanoparticles as well as the transformation to rutile, while the transformation of anatase to rutile was suppressed especially for the sample with the lower titania content (28 mass%). The resulting mesoporous silica-anatase hybrids exhibited higher benzene adsorption capacity (adsorption from water) over those heated at lower temperature, probably due to the dehydroxylation of the silanol group on the pore surface. The photocatalytic decomposition of benzene in water by the present hybrid heated at 1100 °C was efficient as that by P25, a benchmark photocatalyst.

## Introduction

The properties of hybrids of metal oxides, binary or ternary hybrids of different metal oxides, are known to depend on the composition and the heterostructures (the size and shape of each component/domain)^1–3^. Hybrids of metal oxides have been prepared by the multi-step preparation to hybridize hetero-components with pre-synthesized metal oxide nanostructures (building block approach) in addition to the sol–gel reaction to achieve more homogeneously elemental distribution through the materials (bottom-up approach)^4,5^. Macro- to nanoscopic separation of each components in solids^6,7^, elemental doping^8–11^, and core–shell structures^12–15^ are typical hierarchical structures of hybrids of metal oxides, where various amorphous and crystalline domains are involved as a component.

TiO_2_ has been hybridized with porous solids for the application as photocatalysts for environmental purification (degradation of toxic organic compounds in air and water)^16,17^. By the hybridization, molecular recognitive photocatalytic reactions have been achieved^18–20^. The properties of TiO_2_ supported in/on porous materials depend on their crystallinity^21^ and the surface properties^22–25^. Such porous solids as a porous carbon^26^, a metal organic framework^27^, zeolites^28^, clay minerals^29^ and mesoporous silicas^30,31^ have been used to accommodate TiO_2_ particles with varied composition. Among available porous supports, mesoporous silicas have been used extensively for the application as drug carrier, adsorbent and catalysts/photocatalysts due to the attractive characteristic features of mesoporous silicas such as thermal and chemical stability, transparency in the visible to UV–Vis wavelength regions, and well-defined pore shape and size, which influence the heterostructures of the hybrids^31^. The heterostructure of the titania hybridized with mesoporous silicas is used for the photodecomposition of organic pollutants through the possible interactions of the organic pollutants with the pore surface^32^. The introduction of titania nanoparticles in the mesoporous channel has potential in concentrate the organic pollutants, which usually have a low adsorption capability^33^, to decompose the organic pollutants by the active titania nanoparticle, which is exposed to the pore surface. In the present study, the change in the states (location, size etc.) of the TiO_2_ in a mesoporous silica (SBA-15) during the heat treatment was carefully investigated and the changes in the mesostructures was also evaluated at the same time. The hybrid of well-defined anatase nanoparticles^34,35^ immobilized in SBA-15 was used for this objective. The resulting hybrids after the heat treatment exhibited useful adsorptive and photocatalytic properties controlled by the heterostructures and the surface property, which were designed by the host–guest systems and their composition as well as the heat treatment.

## Results and discussion

### Preparation of SBA-15-anatase

**Figure 1:**
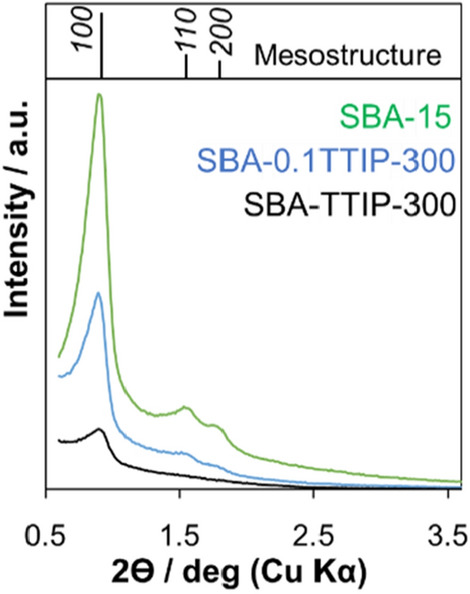
Low angle X-ray powder diffraction patterns of SBA-15 (green), SBA-0.1TTIP-300 (blue) and SBA-TTIP-300 (black).

**Figure 2:**
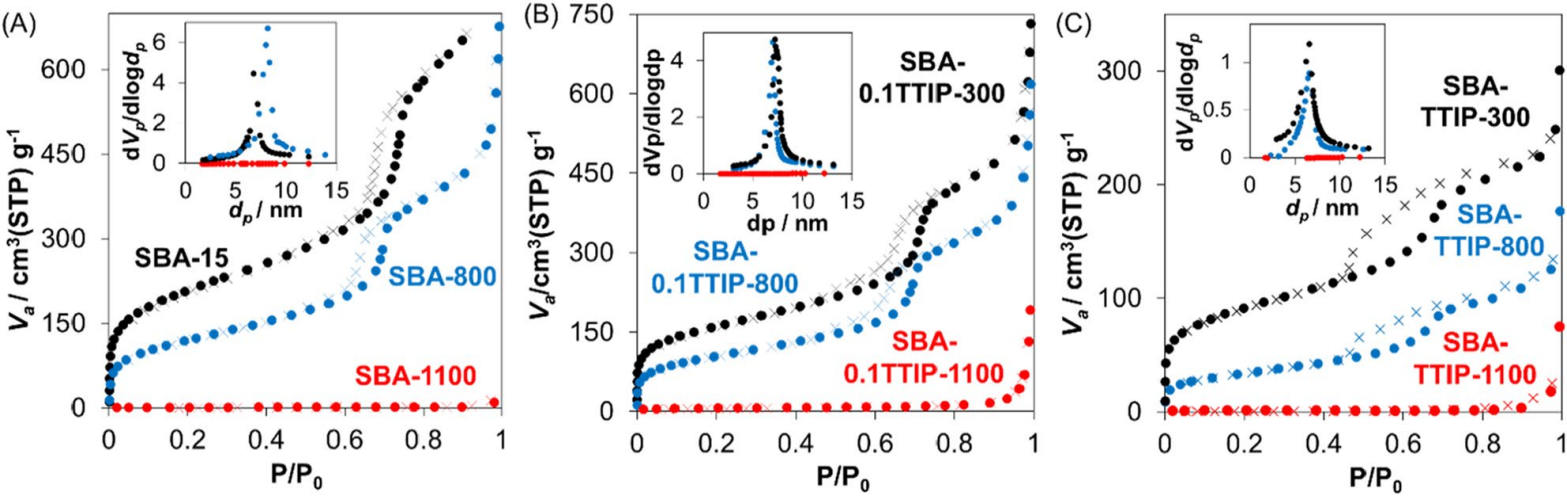
N_2_ adsorption (•)/desorption (x) isotherms and the BJH pore-size distribution (insets) of (**A**) SBA-15, (**B**) SBA-0.1TTIPs and (**C**) SBA-TTIPs before and after the heat treatment at the temperature given in the figure.

The hybrids of TiO_2_ in SBA-15 were prepared by 2 different contents of tetraisopropyl orthotitanate (TTIP) in the solution of isopropyl alcohol (IPA). The composition of SBA-0.1TTIPs and SBA-TTIPs was determined by XRF to be 28 and 65mass% of titania in the hybrids, respectively. The as-prepared SBA-15 and the hybrids were heated at varied temperatures (300, 700, 800, 900, 1000 and 1100 °C) as designed in the name of samples. The XRD patterns of SBA-15, SBA-0.1TTIP-300 and SBA-TTIP-300 are shown in Fig. 1. Reflections due to *(100)*, *(110)* and *(200)* planes of the hexagonally arranged cylindrical pore of SBA-15 were observed, confirming the mesostructure of SBA-15 was retained after the infiltration of titanium oxide precursor and the subsequent crystallization to anatase^36^. The intensities of the reflections were weaker for SBA-0.1TTIP-300 and SBA-TTIP-300, suggesting that the immobilized titania reduced the scattering contrast between the pore and the pore wall of the SBA-15^37^. N_2_ adsorption/ desorption isotherms of SBA-15, SBA-0.1TTIP-300 and SBA-TTIP-300 are shown in Fig. 2. The adsorbed amount of N_2_ was lower for SBA-0.1TTIP-300 and SBA-TTIP-300 if compared with those for SBA-15, suggesting that the mesopores of SBA-15 were filled with titania. Moreover, the crystallite size of anatase in SBA-TTIP-300 was derived from the X-ray diffraction patterns using Scherrer equation to be 8 nm, which was consistent with pore size of the SBA-15 (8 nm) derived from the N_2_ adsorption isotherm of SBA-15 by BJH plot as shown in Table 1.

**Table 1:** The changes in the pore volume and BET surface area of SBA-15, SBA-TTIP and SBA-0.1TTIP by the heat treatment.

Samples	BJH pore size (nm)	Pore volume* (cm^3^ g^−1^ of SiO_2_)	BET surface area** (m^2^ g^−1^ of SiO_2_)
SBA-15	8.0	1.56	730
SBA-800	6.7	0.96	423
SBA-1100	*n.d*	0.05	3
SBA-0.1TTIP-300	7.1	1.49	765
SBA-0.1TTIP-800	7.0	1.25	510
SBA-0.1TTIP-1100	*n.d*	0.38	30
SBA-TTIP-300	*n.d*	1.26	903
SBA-TTIP-800	*n.d*	0.74	334
SBA-TTIP-1100	*n.d*	0.29	16

*BET surface area and pore volume were converted to the value per gram of SiO_2_ using the composition (35 and 72mass%) of SiO_2_ in SBA-TTIP and SBA-0.1TTIP.

*n.d.* not determined.

### Changes of the mesostructure by the heat treatment

**Figure 3:**
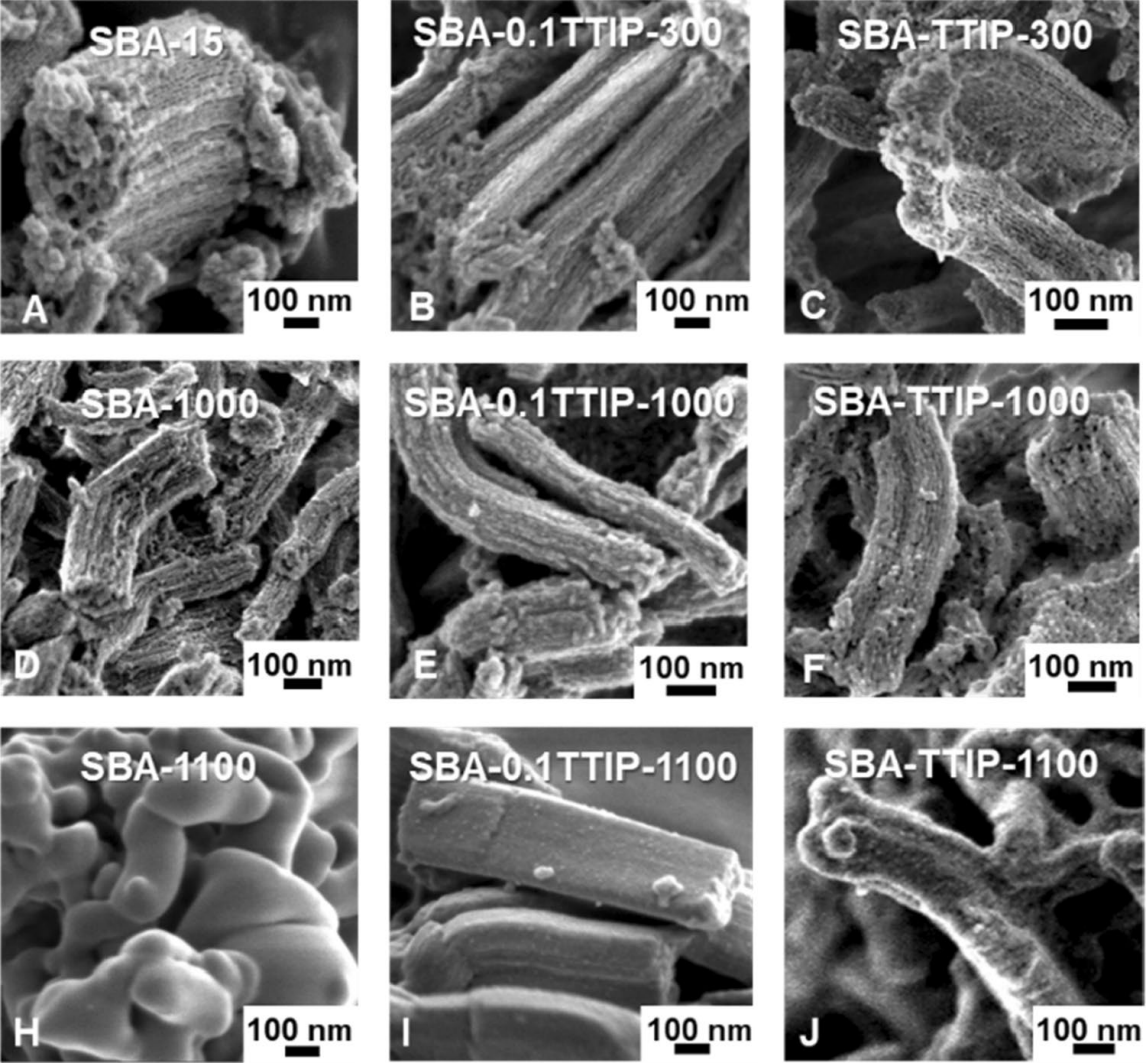
SEM images of (**A**) SBA-15, (**B**) SBA-0.1TTIP-300, (**C**) SBA-TTIP-300, (**D**) SBA-1000, (**E**) SBA-0.1TTIP-1000, (**F**) SBA-TTIP-1000, (**H**) SBA-1100, (**I**) SBA-0.1TTIP-1100 and (**J**) SBA-TTIP-1100.

SEM images of SBA-15, SBA-0.1TTIPs and SBA-TTIPs before and after the heat treatment are shown in Fig. 3. The particle morphology was not affected by the reaction with TTIP, suggesting the successful incorporation of the precursor of titania in the pore. The particle morphology of SBA-15 was retained even after the heat treatment at 1000 °C, while sintering was seen for the sample after the heat treatment at 1100 °C. SBA-0.1TTIP and SBA-TTIP behaved similarly by the heat treatment at 1000 °C, while the particle shapes of SBA-0.1TTIP-1100 and SBA-TTIP-1100 were different from that of SBA-15 heated at the same condition (1100 °C). The bundled tubular shape was maintained with slight modification for SBA-0.1TTIP-1100 and SBA-TTIP-1100.

**Figure 4:**
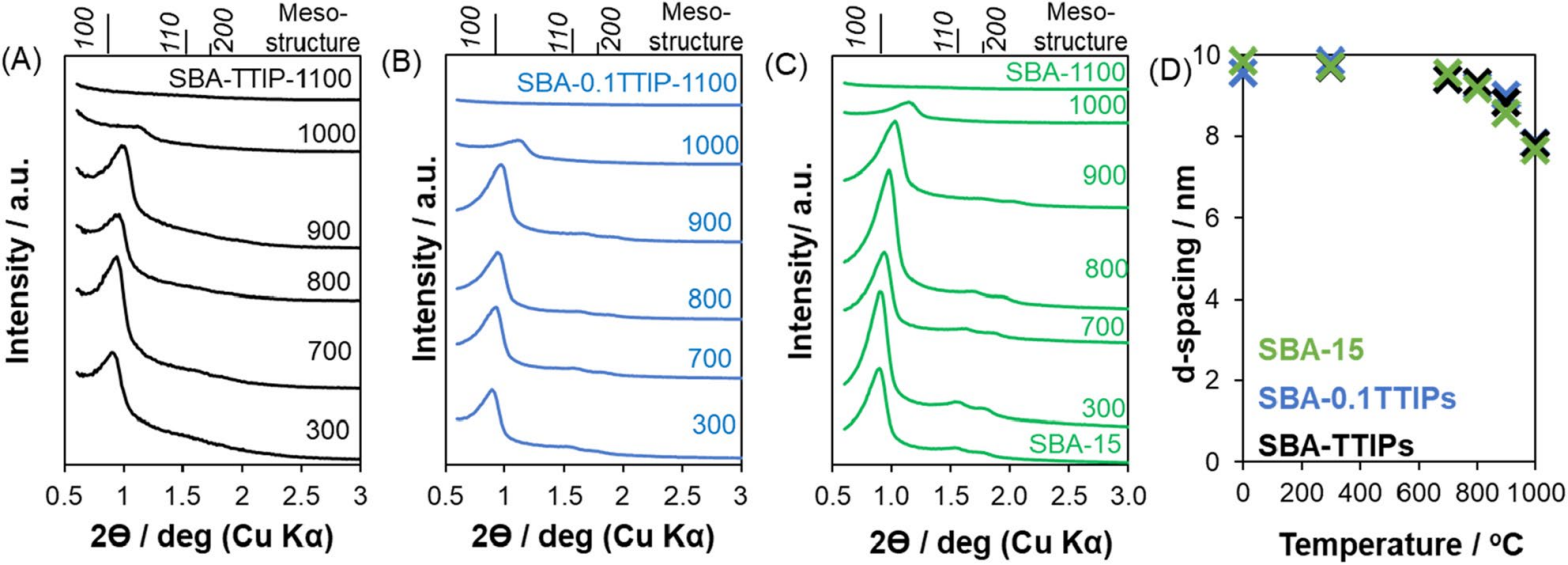
Low-angle X-ray powder diffraction patterns of the heated (**A**) SBA-TTIPs, (**B**) SBA-0.1TTIPs and (**C**) SBA-15, and the changes in *d(100)*-spacing (**D**) of the heated SBA-15 (green), SBA-0.1TTIP (blue) and SBA-TTIP (black), derived from Bragg’s equation as a function of the heating temperature.

The changes in the low angle X-ray diffraction patterns of SBA-TTIP, SBA-0.1TTIP and SBA-15 by the heat treatment are shown in Fig. 4A–C, respectively. The mesostructure of SBA-15 changed in accordance with the morphological change. The reflection due to *(100)* became broad after the heat treatment and disappeared for the samples heated at 1100 °C. The changes of *d(100)*-spacing are summarized in Fig. 4D. Pore size and pore volume were derived by BJH method, which represents the relationship between critical condensation pressure and mesopore size with the assumption of cylindrical-shaped pores^38–40^, as shown in Fig. 2 inset. The changes of BJH pore size, pore volume and BET surface area, which were derived from the N_2_ adsorption isotherms, of the heated SBA-15, SBA-0.1TTIPs and SBA-TTIPs are summarized in Table 1. After the heat treatments, the porosity of all the samples decreased (the densification of mesoporous silica), meaning that the pore was collapsed, which are consistent with the XRD and SEM results.

### Structural and size changes of the occluded titania particles

**Figure 5:**
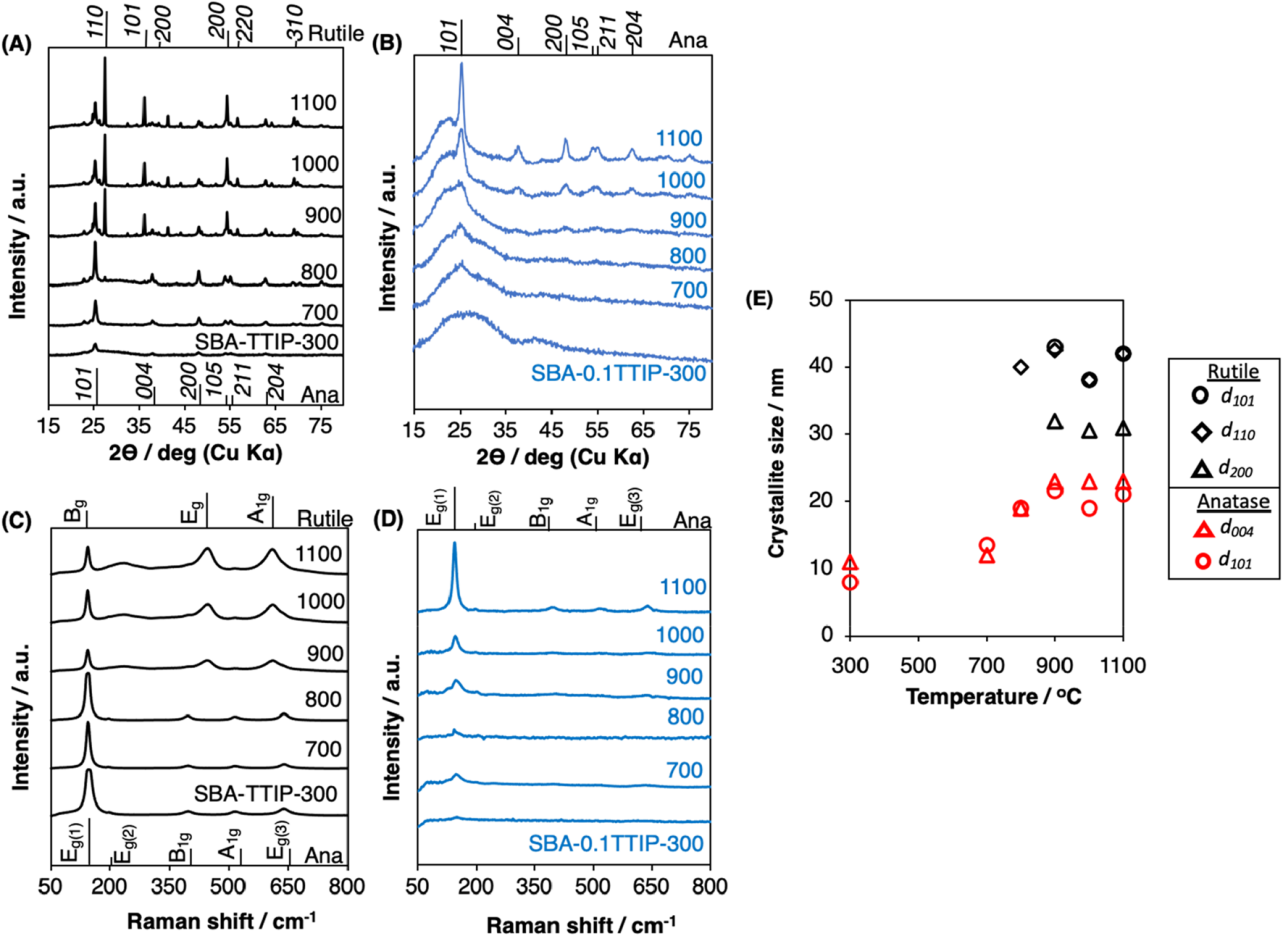
X-Ray powder diffraction patterns of the heated (**A**) SBA-TTIPs and (**B**) SBA-0.1TTIPs, Raman spectra of the heated (**C**) SBA-TTIPs and (**D**) SBA-0.1TTIPs (Ana shorted from Anatase) and crystallite size of titania in (**E**) SBA-TTIPs after the heat treatment at designed temperatures.

**Figure 6:**
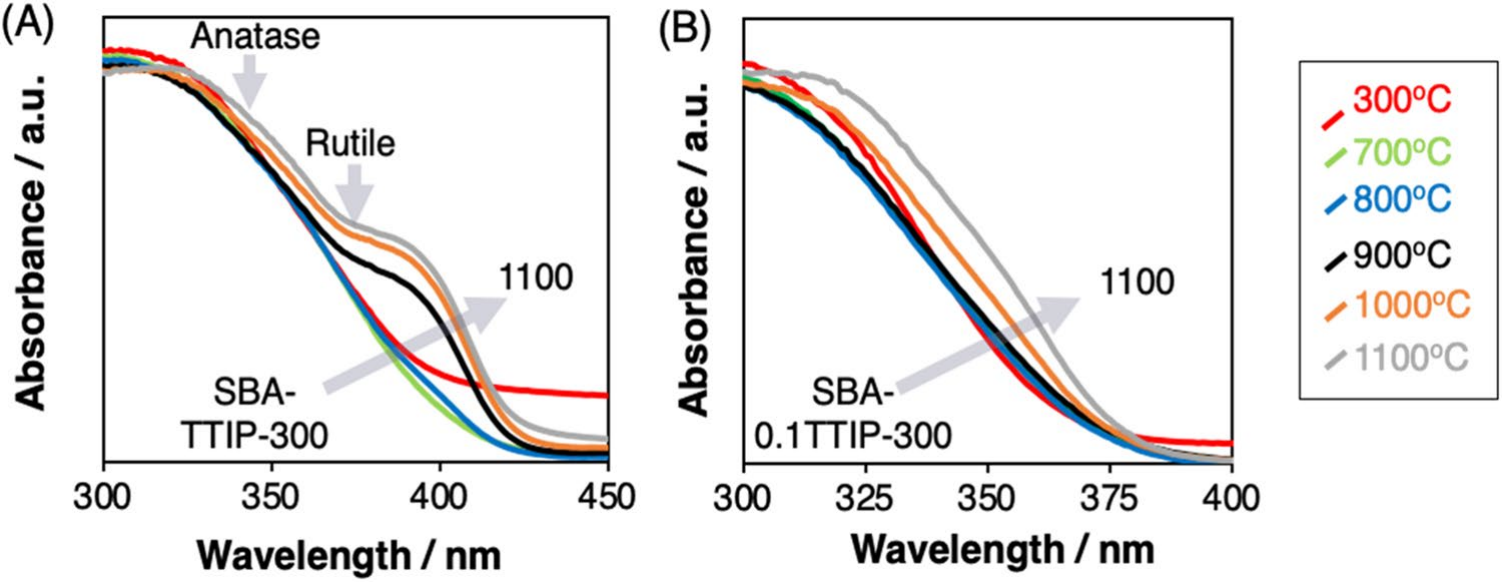
Diffuse reflectance spectra of the heated (**A**) SBA-TTIPs and (**B**) SBA-0.1TTIPs (the spectra due to anatase and rutile are indicated by the arrows).

**Figure 7:**
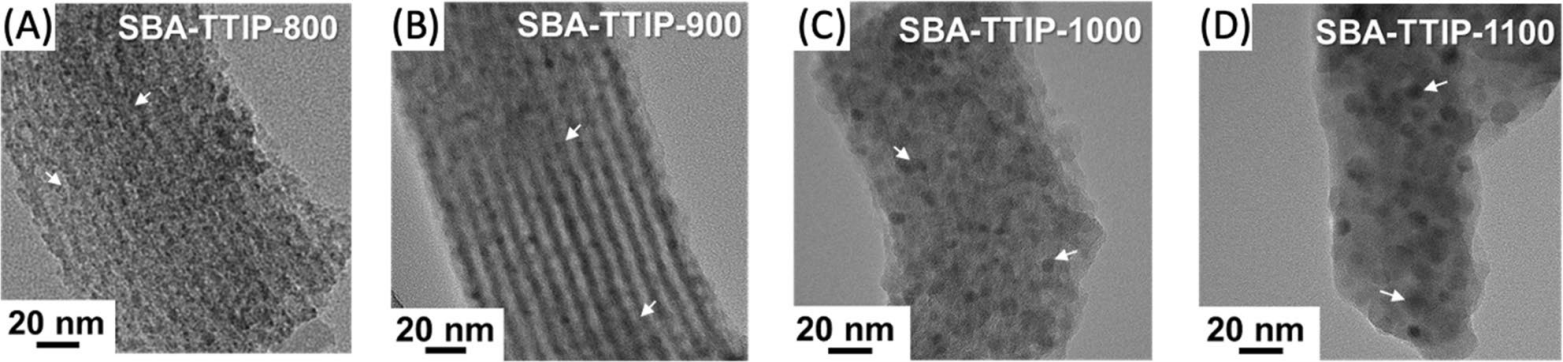
TEM images of SBA-TTIP heated at (**A**) 800, (**B**) 900, (**C**) 1000 and (**D**) 1100 °C (the titania particles are shown by the arrows).

The changes of the XRD patterns and diffuse reflectance spectra of the SBA-TTIP by the heat treatment at varied temperatures are shown in Figs. 5A and 6A. As reported previously^34,35^, the amorphous precursor derived from TTIP in the mesopore crystallized into anatase by the heat treatment at 300 °C. The transformation of anatase to rutile was seen for the samples heated at above 700 °C, while, even after the heat treatment at 1100 °C, the anatase phase co-existed. The crystallite size of anatase and rutile as a function of the heat treatment are summarized in Fig. 5E. The crystallite size of anatase along *(101)* and *(004)* planes increased similarly (from 8 to 20 nm and from 10 to 22 nm for *(101)* and *(004)*, respectively) by the heat treatments at 300 °C to 1100 °C. The crystallite size of rutile for SBA-TTIP-800 was 40 nm, which was two-fold of the crystallite size of anatase in the same sample, suggesting that the fusion/growth of the anatase particles occurred to form rutile during the heat treatment^41^. The population of the rutile phase relative to the anatase phase increased by the heat treatment at higher temperatures as seen by the XRD patterns (Fig. 5A) and Raman spectra (Fig. 5C). TEM images of SBA-TTIPs are shown in Fig. 7. The deformation of the mesostructure and the crystallite growth of anatase and rutile particles after the heat treatments at 800–1100 °C were shown by the TEM images, which were consistent with the XRD results.

**Figure 8:**
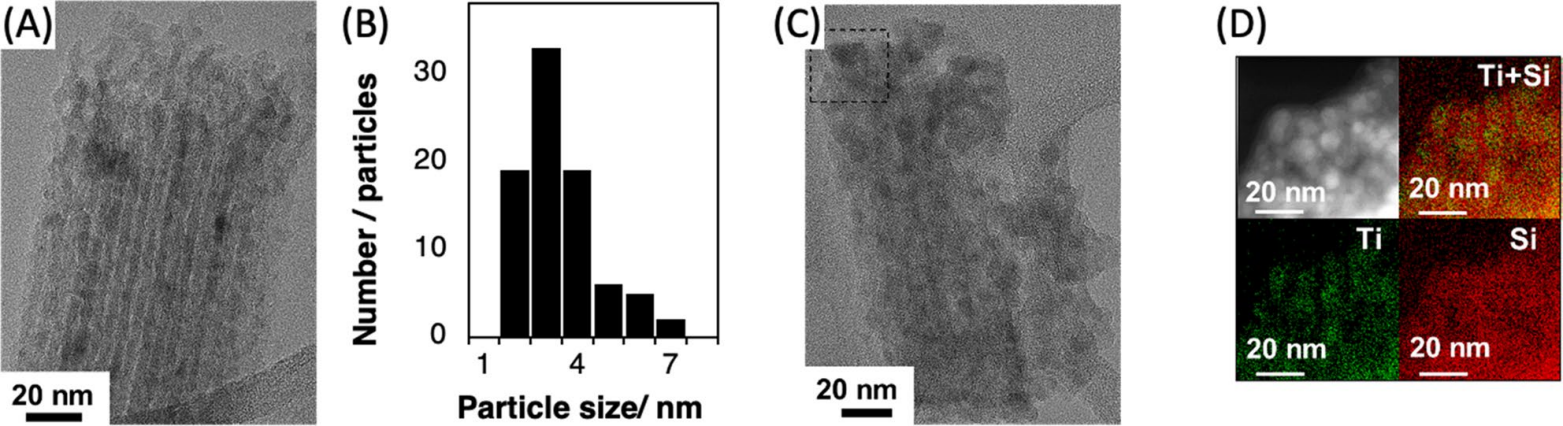
TEM image of (**A**) SBA-0.1TTIP-800 and (**C**) SBA-0.1TTIP-1100, (**B**) the particle size distribution of the anatase in SBA-0.1TTIP-800 derived from the TEM and EDS elemental analysis (**D**) of SBA-0.1TTIP-1100.

The XRD patterns and the Raman spectra of the SBA-0.1TTIP after the heat treatment at varied temperatures are shown in Fig. 5B,D, respectively. Reflections from crystalline titania in the SBA-0.1TTIPs heated at the temperature lower than 900 °C were hardly detected in the XRD patterns probably due to the low titania content and smaller particle size, while Raman spectra indicated the presence of anatase. The Eg(1) peak due to anatase^42^ at 144 cm^−1^ was observed for all the heated SBA-0.1TTIPs. The transformation to rutile was not seen for SBA-0.1TTIPs even after the heat treatment at 1100 °C, in contrast to the results for SBA-TTIP. Diffuse reflectance spectra of the heated SBA-0.1TTIP are shown in Fig. 6B, where absorption due to anatase was seen with the absorption edges at 365–369 nm for the samples heated at 300–800 °C. These results indicated that the particle size of the anatase unchanged during the heat treatments at 300–800 °C. The TEM images of SBA-0.1TTIPs by the heat treatments at 800 to 1100 °C are shown in Fig. 8A,C. The average size of the anatase particle was 3 nm as determined from the particle size distribution of the anatase particles in SBA-0.1TTIP-800 (Fig. 8B), which was derived from the TEM image (Fig. 8A). By the heat treatment at 1100 °C, the mesostructure of SBA-0.1TTIP was collapsed and the anatase particles grew from 3 to 8 nm as shown by the XRD pattern, nitrogen adsorption/desorption isotherms and the TEM image. The EDS elemental mapping of SBA-0.1TTIP-1100 is shown in Fig. 8D, confirming the growth of anatase particles. Considering the titania content (28mass%) of SBA-0.1TTIP-1100, it was thought that the anatase particles were grown to be located on the particle surface composed of the densified silica.

**Figure 9:**
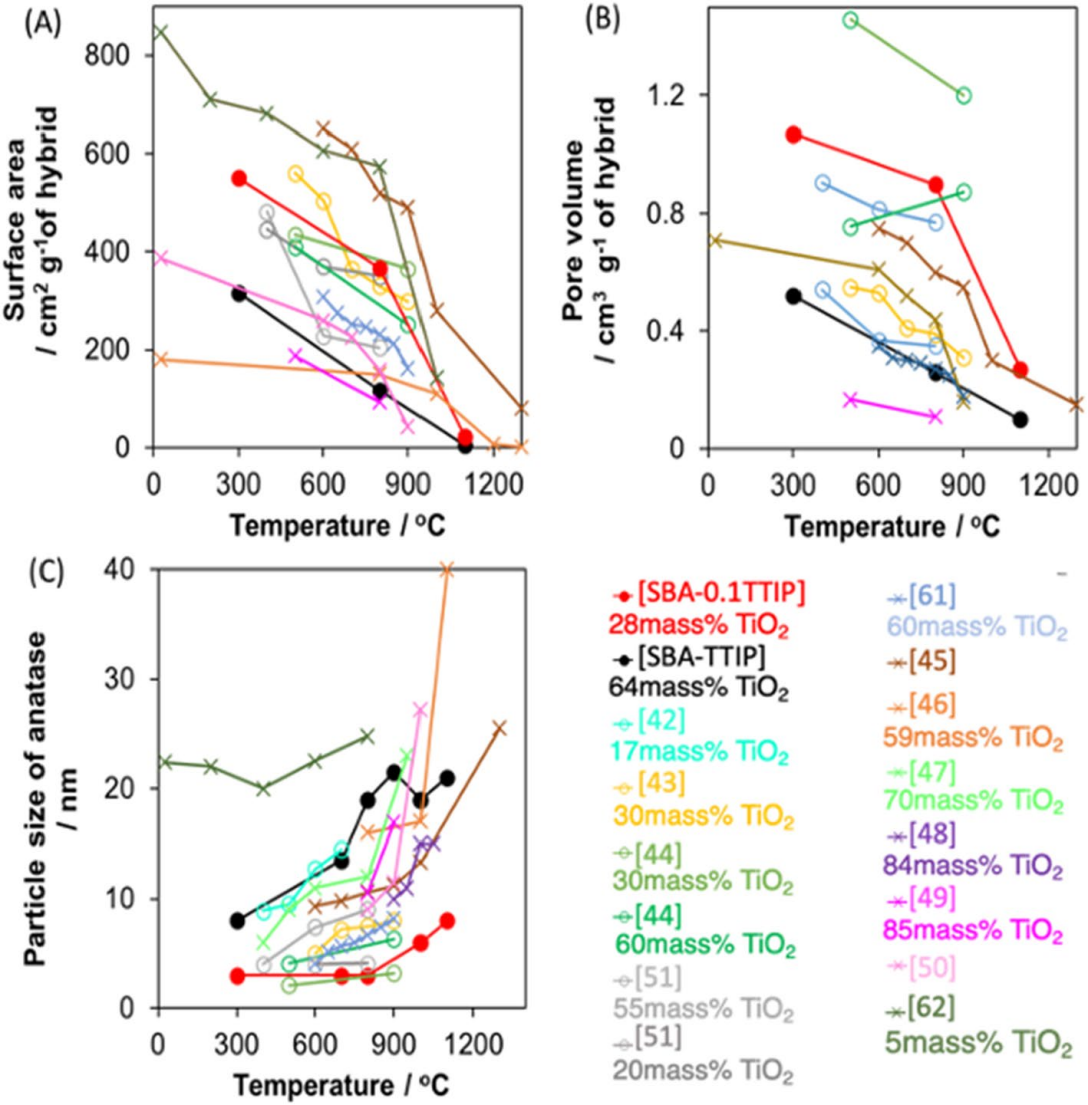
The relationship between (**A**) BET surface area, (**B**) pore volume and (**C**) particle size of anatase as a function of the heat treatment temperature where -o- represents those prepared by impregnation method and -x- represents those prepared by co-condensation method.

**Scheme 1:**
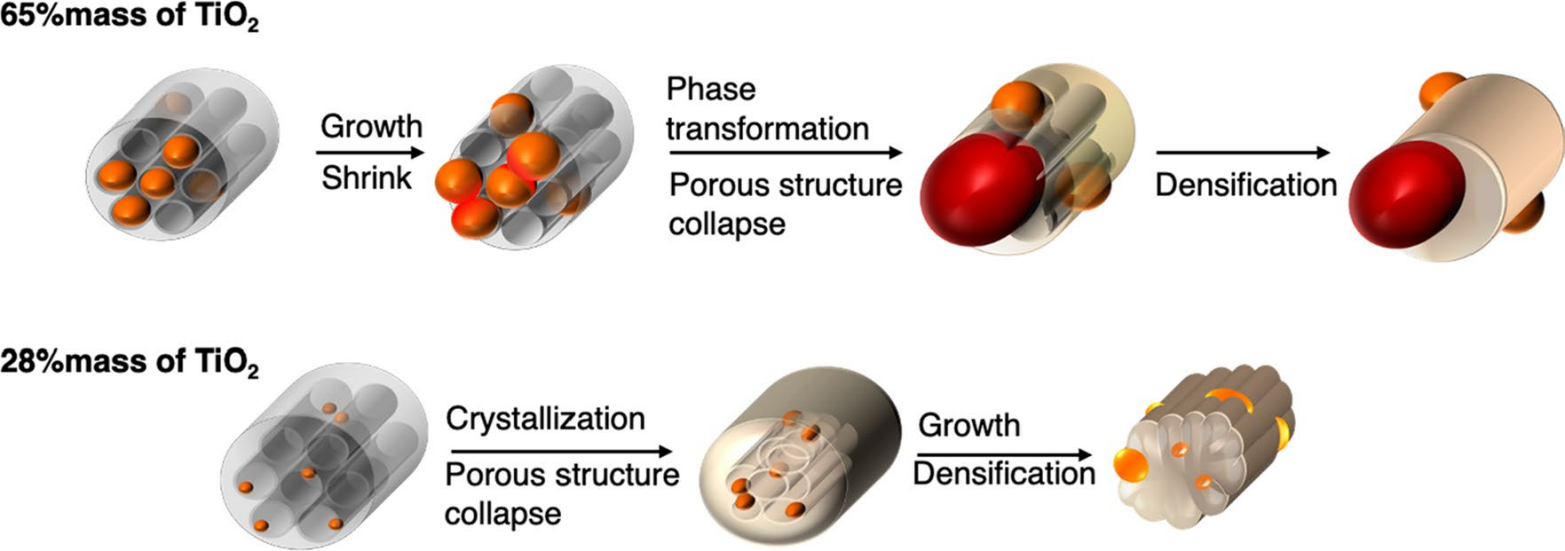
The proposed heterostructural changes of SBA-TTIP and SBA-0.1TTIP by the heat treatment. This scheme was created by using Microsoft PowerPoint version 16.43 (Product ID:02954–080-106679). *Orange represents anatase phase and red represents rutile phase.

The phase transformation of bulk anatase to rutile occurs at 600 °C under atmospheric pressure^43^. The retardation of the phase transformation has been examined by hybridizing titania with silica, where the interactions of titania and silica are thought to suppress the growth of anatase and the formation of rutile^44–46^. The doped silica particle in the mesoporous titania (with 1–20mass% silica) was reported to hinder the growth of anatase by the heat treatment (below 800 °C)^47–52^. The doped silica particle expelled to the surface of anatase resulting in the collapse of mesostructure and the aggregation of anatase nanodomain upon the heat treatments over 1000 °C, affecting the growth of the anatase particles. This correlation was plotted in Fig. 9C. In addition, mesoporous silica-anatase hybrids have been prepared by impregnation method^44–46,53^. The location of the anatase particle may not well controlled in the mesoporous silica. The significant decreases in the surface area and the pore volume of the reported hybrids^45,53^ (Fig. 9A,B) indicated the collapse of their mesostructure at 600 °C due to the growth of anatase particle on the external surface of the mesoporous silica. The anatase on the mesoporous silicas (with 17 and 30mass% of titania)^44,45^ grew as the samples in this work (SBA-TTIPs with 65%mass of titania) by the heat treatments, that led the phase transformation to rutile at 900 °C in the reported hybrid^45^. In the present study, the interactions between mesoporous silica and the anatase nanoparticles were thought to be concerned for the suppressed transformation of anatase to rutile. For SBA-0.1TTIP, anatase particle was smaller than those in SBA-TTIP and the distance between adjacent anatase particles was expected to be longer than that in SBA-TTIP. These parameters were also thought to affect the aggregation, fusion and crystal growth of anatase and the transformation to rutile. These results suggested the heterostructural changes of SBA-TTIP and SBA-0.1TTIP by the heat treatment as shown in Scheme 1.

**Figure 10:**
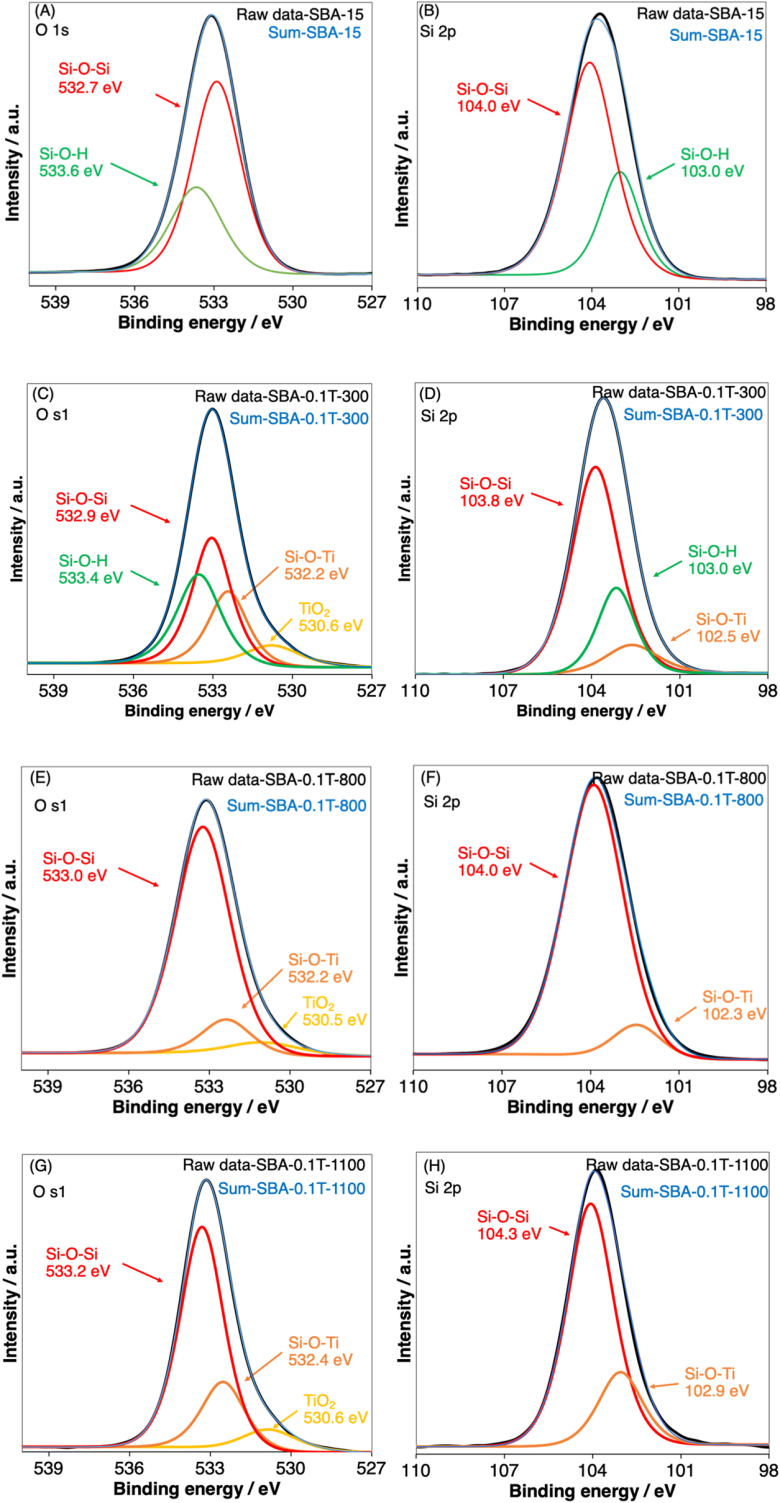
XPS spectra of (**A**,**B**) SBA-15, (**C**,**D**) SBA-0.1 T-300, (**E**,**F**) SBA-0.1 T-800 and (**G**,**H**) SBA-0.1 T-1100 which respected with O1s and Si2p.

The structural changes of the hybrids by the heat treatment was further investigated by X-ray photoelectron spectroscopy (XPS). The XPS spectra and the atomic contents of Si and O, estimated from the XPS spectra of SBA-15 and the heated SBA-0.1TTIPs (at 300, 800 and 1100 °C) are shown in Fig. 10 and Table 2, respectively. The O1s and Si2p peaks of SBA-15 (Fig. 10A,B) were fitted with 2 peaks ascribable to Si–O–Si and Si–O–H in descending by area of the peaks^54^. The peak due to Si–OH indicated the presence of silanol group^17^. The XPS spectra of SBA-0.1TTIP-300 are shown in Fig. 10C,D, where the O1s peak was fitted with 4 peaks of Si–O–Si, Si–O–H, Si–O–Ti and TiO_2_ associated with those of Si2p peak^55^. The Si–OH peak, which corresponds to silanol group, was seen at the binding energy of 533.4 eV (for O1s) and 103.0 eV (for Si2p). Whereas, in the XPS spectra of SBA-0.1TTIP-800 (Fig. 10E,F) and SBA-0.1TTIP-1100, (Fig. 10G,H) peaks due to silanol group were not seen. These results suggested that the silanol group disappeared by the dehydroxylation to the siloxane linkage by the heat treatment over 800 °C.

**Table 2:** Quantitative evaluation of atomic contents of O and Si in the samples from XPS analysis.

Samples	Atomic content (%)		Assignment
	O1s	Si2p	
SBA-15	45.7	24.5	Si–O–Si
	20.0	9.8	Si–O–H
SBA-0.1TTIP-300	42.8	22.2	Si–O–Si
	15.7	9.2	Si–O–H
	7.1	3.0	Si–O–Ti
SBA-0.1TTIP-800	55.8	31.5	Si–O–Si
	8.8	3.9	Si–O–Ti
SBA-0.1TTIP-1100	47.9	28.7	Si–O–Si
	14.8	8.6	Si–O–Ti

The XPS analysis of SBA-0.1TTIP-1100 (Fig. 10G,H) demonstrated that the atomic content associating to Si–O–Ti was larger over those of SBA-0.1TTIP-300 and SBA-0.1TTIP-800. Since XPS is a surface sensitive analysis, the higher content of Si–O–Ti suggested that the location of the TiO_2_ on the surface. These XPS results supported the proposed change of the heterostructure of SBA-0.1TTIPs by the heat treatment as shown in Scheme 1.

### Adsorption and photocatalytic decomposition of benzene

**Figure 11:**
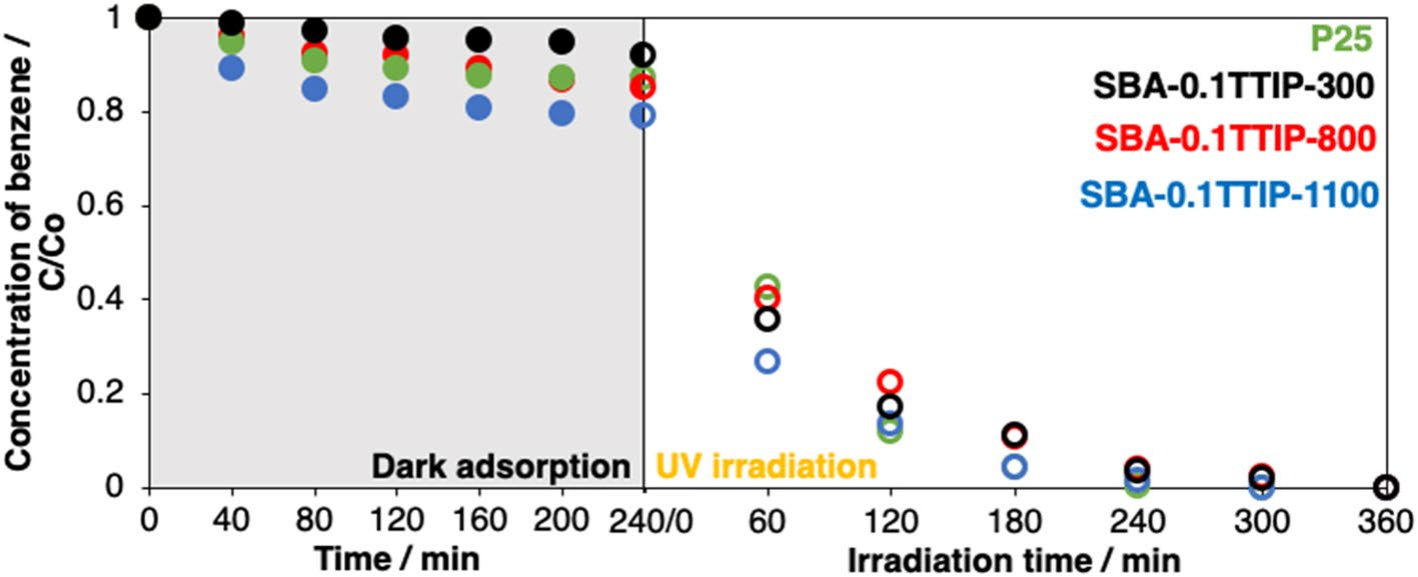
Changes in the relative concentration (C/C_0_) of benzene under the dark adsorption and the UV irradiation. The initial concentration (C_0_) of benzene was 300 ppm.

The hybrids of metal oxide photocatalysts with porous supports have been applied for the treatment of the pollutants^56–59^. The photocatalytic decomposition of benzene in water by UV irradiation by SBA-0.1TTIP-300, SBA-0.1TTIP-800, SBA-0.1TTIP-1100 and a commercially available photocatalyst (P25) was examined. The adsorption of benzene on the hybrids in the dark was followed by the changes in the concentration of benzene in the solution as a function of the contact time as shown in Fig. 11. The adsorption leached the equilibrium within 4 h and the adsorbed amounts of benzene on SBA-0.1TTIP-1100, SBA-0.1TTIP-800 and SBA-0.1TTIP-300 were 0.14, 0.07 and 0.04 g of adsorbed benzene/g of the hybrids, respectively. Even though the BET surface area decreased by the heat treatment at higher temperature (Table 1), larger amount of benzene was adsorbed for the samples heated at higher temperature. It was thought that the silanol and titanol groups on the surface of the hybrids were dehydroxylated by the heat treatment to give hydrophobic surface for the adsorption of benzene. After the adsorption of benzene on the photocatalysts leached the equilibrium, UV light was irradiated to the reactor. The photocatalytic decomposition of benzene by P25, SBA-0.1TTIP-300, SBA-0.1TTIP-800 and SBA-0.1TTIP-1100 was followed by the change in the concentration as a function of the irradiation time (Fig. 11). The changes of the concentration of benzene were fitted with logarithmic expression as a first-order kinetics and the rate constants and the experimental errors derived are shown in Table 3. The benzene was completely decomposed within 6 h for all the tested samples. The rate of the decomposition of benzene by SBA-0.1TTIP-1100 was similar to that by P25. The mineralization of benzene on TiO_2_ photocatalyst is known as 2 routes via phenol or muconaldehyde, then, further oxidized to CO_2_^60^. Phenol was detected as the main product together with the smaller amount of catechol in the present study and finally decomposed to CO_2_, irrespective of the photocatalysts.

**Table 3:** Apparent first-order rate constant *k* for the decomposition of benzene and their corresponding global correlation coefficient *R*^2^ value.

	Samples			
	P25	SBA-0.1TTIP-300	SBA-0.1TTIP-800	SBA-0.1TTIP-1100
*k* (min^−1^)	0.0075	0.0058	0.0056	0.0075
*R*^2^	0.99	0.99	0.99	0.99

The structural deformation of mesoporous silica-anatase hybrids by the heat treatment at the temperatures over 800 °C was reported to suppress the photocatalysis performance on the degradation of methylene blue and the H_2_ evolution from water on the anatase nanoparticle supported in SBA-15 and SiO_2_ foam, respectively^45,46^. Efficiency of the oxidation of ethylene on the TiO_2_/SiO_2_ photocatalyst prepared by a sol–gel reaction was reduced after the heat treatment above 350 °C, which was ascribed to the decrease in the surface area^61^. The heat treatment also affects the crystalline form, crystallinity, particle size, oxygen defect and so on, which can influence the electronic structure and the geometric structure of TiO_2_ nanoparticles. In the present study, the immobilized anatase particle in the mesoporous silica heated at 1100 °C showed the enhancement in the photocatalytic activity on the decomposition of benzene, and the efficiency was higher than those achieved by the hybrids heated at lower temperature and comparable with the well-known titania photocatalyst (P25). The elimination of the bridged OH groups, which was confirmed by the XPS analysis, by the heat treatment seemed to lead the hydrophobic surface, which promoted the direct adsorption of the hydrophobic molecules that were then oxidized as reported by Nosaka et al.^62^. These finding suggests the new concept of the structural design from the mesoporous silica-nanoparticle hybrids by the heat treatment. Further systematic study using hybrids composed of mesoporous silicas with varied pore size and nanoparticles with varied contents combined with varied heat treatment condition is worth investigating to control the photocatalytic reactions in more detail.

## Conclusions

Heterostructural transformation of mesoporous silica (SBA-15 with the BJH pore size of 8 nm) containing anatase nanoparticles in the pore with different titania contents (28 and 65 mass%) was investigated by the heat treatment in air up to 1100 °C. The anatase particles fused and transformed to rutile at 800 °C for the sample with 65%mass TiO_2_, whereas the size of anatase nanoparticle and mesostructure of the samples with 28%mass TiO_2_ were unchanged at 800 °C and maintained anatase nanoparticle even after the heat treatment at 1100 °C. The mesostructured of SBA-15 was collapsed by the heat treatment over 1000 °C. The samples with 28%mass TiO_2_ after the heat treatment at 800 and 1100 °C exhibited higher benzene adsorption capacity due to the hydrophobic surface caused by the dehydroxylation of the surface silanol group during the heat treatment. The sample heated at 1100 °C showed the efficient photocatalytic decomposition of benzene in water by UV irradiation with the efficiency comparable to a benchmark photocatalysts, P25, thanks to the crystallization of anatase nanoparticles and the heterostrcutural transformation, which contributed the growth of anatase nanoparticle on the external surface of the collapsed SBA-15 particle.

## Experimental section

### Materials

Tetraethyl orthosilicate (abbreviated as TEOS), tetraisopropyl orthotitanate (abbreviated as TTIP) and a poly(ethylene glycol)-block-poly(propylene glycol)-block-poly(ethylene glycol) block copolymer (Pluronic P123, abbreviated as P123) were purchased form Aldrich. Hydrochloric acid was purchased from Tokyo Chemical Ind. Co. All the reagents were used without further purification. Water was purified by using Milli-Q system (> 18 MΩ cm, Millipore).

### Preparation of SBA-15

SBA-15 was synthesized by the reported method^36^ as follows, 10.0 g of P123 was dissolved in 75 g of water and 300 g of 2 M HCl solution using magnetic stirrer at 35 °C. To which solution was added 21.2 g of TEOS and the mixture was vigorously stirred at 25 °C for 20 h. The mixture was aged at 120 °C for 1 day without stirring. The product was collected by filtration, washed with water, and dried at 25 °C. P123 was removed by the calcination of the product at 500 °C for 6 h.

### Introduction of TiO_2_ into SBA-15 and the heat treatment

TiO_2_ was introduced into SBA-15 by the method according to the previous reports^34,35^ as follows; SBA-15 was dehydrated at 120 °C for 3 h under a reduce pressure, and after cooling down to room temperature under the reduced pressure (1 bar), 45 ml of TTIP or 4.5 ml of TTIP in IPA (1:9 volume ratio) was injected into 1 g of the dehydrated SBA-15 under the reduced pressure. The mixtures were magnetically stirred at room temperature for 1 day. The solids were separated by the centrifugation at 4000 rpm for 15 min and the remaining liquid in the sediment was removed by absorbing with a cellulose filter paper. For the hydrolysis and the condensation of the introduced TTIP, the products were exposed to HCl vapor at room temperature for 1 day. The samples prepared from neat TTIP and the IPA solution of TTIP were designed as SBA-TTIP and SBA-0.1TTIP, respectively. The samples were heated at 300, 700, 800, 900, 1000 and 1100 °C in air for 3 h at the heating rate of 5 °C/min. The heated samples were designed as SBA-XTTIP-Y where Y is the heat treatment temperature.

### Characterizations

The X-ray powder diffraction patterns of the products were recorded using Bruker New D8 Advance equipped using CuKα radiation. Scanning electron micrographs (SEM) were obtained on a JEOL JSM-7610F field emission scanning electron microscope. Prior to the measurements, the samples were coated with platinum (the thickness of 4 nm). The elemental distribution and crystallite size were examined by FEI-TF20 field emission transmission electron microscope. The Ti/Si ratio was determined on a wavelength-dispersive X-ray fluorescence spectrometer (WDXRF, Bruker S8 Tiger). The chemical surface analysis was evaluated by X-ray photoelectron spectroscopy (XPS, JPS-9010MC, JEOL) with Mg–Ka radiation source (hν = 1253.6 eV). N_2_ adsorption/desorption isotherms were obtained at − 196 °C on a Belsorp Mini instrument (MicrotracBEL Corp). Prior to the measurement, the samples were dehydrated at 120 °C under vacuum for 2 h. The surface area was calculated by the Brunauer–Emmett–Teller (BET) method using a linear plot in the range of P/P_0_ = 0.05–0.20^63^. Pore size distributions were derived from the N_2_ adsorption isotherm by BJH method^31^. The diffuse reflectance spectra were obtained by using UV spectrometer (Perkin Elmer Lambda 1050 UV/Vis/NIR Spectrophotometer) with an integrate sphere.

### Adsorption and photocatalytic reaction of benzene

The photocatalytic decomposition of benzene using SBA-0.1TTIPs was carried out in a quartz tubular reactor with inner tube to place 400 W Hg lamp. The sample (100 mg) was suspended in 220 ml of aqueous solution of benzene (300 ppm). The reactor was kept at around 10 °C with cooling water circulation during the irradiation. The suspension was sampled every 30 min. The concentration of benzene was determined by an HPLC system (1260 Infinity, Agilent Technologies) with a 4.6 × 250 mm column (Inertustain C18 5um, GL-Sciences), after the catalyst was separated by ultracentrifugation at 20,000 rpm for 20 min at 10 °C. The HPLC mobile phase was a mixture of methanol and water (volume ratio of 1:1).
